# Patients with IVF complicated by moderate-to-critical OHSS experience increased thrombosis, GDM and neonatal NICU admission but slightly shorter gestation compared with matched IVF counterparts: A retrospective Chinese cohort study

**DOI:** 10.1186/s12958-020-00678-w

**Published:** 2021-01-13

**Authors:** Linli Hu, Rui Xie, Mengying Wang, Yingpu Sun

**Affiliations:** 1 Center for Reproductive Medicine, Henan Key Laboratory of Reproduction and Genetics, The First Affiliated Hospital of Zhengzhou University, Jianshe Dong Road, Henan 450052 Zhengzhou, People’s Republic of China; 2 Center for Reproductive Medicine, Affiliated Shenzhen Maternity and Child Healthcare Hospital, Southern Medical University, Fuqiang Road, Guangdong 518000 Shenzhen, People’s Republic of China

**Keywords:** OHSS, Pregnancy outcomes, Neonatal outcome, In vitro fertilization, Infertility

## Abstract

**Background:**

Ovarian hyperstimulation syndrome (OHSS) is a common disease during controlled ovarian hyperstimulation treatment. However, the obstetric and neonatal outcomes of this group of patients are unknown. The aim of this study was to explore the effects of late moderate-to-critical OHSS on obstetric and neonatal outcomes.

**Methods:**

This prospective observational study included 17,537 patients who underwent IVF/ICSI-fresh embryo transfer (ET) between June 2012 and July 2016 and met the inclusion criteria, including 7,064 eligible patients diagnosed with clinical pregnancy. Ultimately, 6,356 patients were allocated to the control group, and 385 patients who were hospitalized and treated at the center for late moderate-to-critical OHSS were allocated to the OHSS group. Then, propensity score matching analysis was performed, matching nine maternal baseline covariates and the number of multiple gestations; 385 patients with late moderate-to-critical OHSS were compared with a matched control group of 1,540 patients. The primary outcomes were the live birth rate, preterm delivery rate, miscarriage rate, gestational age at birth (weeks), obstetric complications and neonatal complications.

**Results:**

The duration of gestation in the matched control group was significantly higher than that in the OHSS group. The live birth delivery rate did not significantly differ between the OHSS and matched control groups. The incidence rates of the obstetric complications venous thrombosis (VT) and gestational diabetes mellitus (GDM), neonatal complications and the number of neonates admitted to the NICU were significantly higher in the OHSS group than in the matched control group.

**Conclusions:**

Pregnant women undergoing IVF with fresh ET whose course is complicated by late moderate-to-critical OHSS appear to experience shortened gestation and increased obstetrical and neonatal complications compared with matched controls whose course is not complicated by OHSS. However, the live birth rate, average neonatal weight, and incidence rates of premature delivery, miscarriage, early abortion, hypertensive disorder of pregnancy (HDP), placenta previa (PP), intrahepatic cholestasis of pregnancy (ICP), and low neonatal birth weight (LBW) did not differ significantly between the two groups.

## Introduction

The widespread use of assisted reproductive technology (ART) in the clinic to enhance the oocyte number has increased the prevalence of ovarian hyperstimulation syndrome (OHSS) [1, 2]. The etiopathogenesis of OHSS remains unclear, but hCG, VEGF, angiotensin, and interleukin seem to be the key players in OHSS. These factors increase capillary permeability and cause blood volume reduction, blood concentration, liver function damage, kidney function damage, water and electrolyte disorders, thrombosis, acute respiratory distress syndrome and other clinical manifestations, and the condition can be life-threatening [3–5]. Clinical studies indicate that the incidence of moderate-to-severe OHSS is approximately 2–3%, and milder forms may develop in up to 20– 30% of all in vitro fertilization (IVF) patients [5]. The clinical symptoms of OHSS are highly variable and difficult to precisely classify, and uniform standards are lacking, rendering accurate clinical data collection and unified classification difficult.

Recently, numerous reports concerning the prevention and treatment of OHSS have been published. However, the pregnancy outcomes of OHSS are poorly understood and remain controversial likely because the impact of late OHSS on pregnancy outcomes is difficult to predict [6–10]. Earlier studies have demonstrated that pregnancy and abortion rates are significantly increased among OHSS patients and that these patients are more likely to develop adverse pregnancy outcomes, such as abortion, growth restriction, hypertensive disorder of pregnancy (HDP), gestational diabetes mellitus (GDM) and low neonatal birth weight (LBW) [1, 11–17]. In addition, one study reported that the hospitalization of OHSS patients who underwent IVF was not conducive to pregnancy or continued pregnancy [18]. The aim of this study was to investigate the effects of late moderate-to-critical OHSS on pregnancy and neonatal outcomes in pregnancies conceived following IVF/ICSI fresh embryo transfers.

## Materials and methods

This prospective observational study was approved by the Institutional Review Board of the First Affiliated Hospital of Zhengzhou University and the Institutional Ethics Committee Review Board of the First Affiliated Hospital of Zhengzhou University, Zhengzhou University (Scientific research-2019-LW-046). This study was retrospectively registered on May 26th, 2017 (ChiCTR-1,800,014,655).

The inclusion criteria in this study were as follows: IVF/ICSI-fresh embryo transfer (ET) and an age ≤ 40 years. The exclusion criteria included the following: preimplantation genetic testing (PGT), donor sperm and donor oocyte, female age > 40 years, missing clinical data, sperm extraction method for percutaneous epididymis/testicular puncture/testicular biopsy (PESA/TESA/TESE), parental chromosomal abnormalities, Mullerian tube abnormalities (uterine malformations), and other diseases that may affect pregnancy outcomes, including an adverse pregnancy history, diabetes, hyperprolactinemia, hypothyroidism, hyperthyroidism, cervix postoperative conization, pituitary tumor, pituitary microadenoma, premature ovarian failure, a history of pelvic tuberculosis, a history of rheumatic immune system disease, and a history of psychiatric diseases.

The classification criteria for OHSS were as follows: early-onset OHSS indicated the occurrence of OHSS no later than 9 days after the hCG injection, and late OHSS indicated the occurrence of OHSS generally no earlier than 10 days after the hCG injection [5, 15].

The classification of OHSS symptoms were as follows: mild: abdominal distension/discomfort, mild nausea/vomiting, mild dyspnea, diarrhea, enlarged ovaries, and no important alterations; moderate: mild features, ultrasonographic evidence of ascites, hemoconcentration (Hct > 41%), and elevated WBC (> 15,000 mL); severe: mild and moderate features, clinical evidence of ascites, hydrothorax, severe dyspnea, oliguria/anuria, intractable nausea/vomiting, severe hemoconcentration (Hct > 55%), WBC > 25,000 mL, CrCl < 50 mL/min, Cr > 1.6 mg/dL, Na + < 135 mEq/L, K + > 5 mEq/L, and elevated liver enzymes; critical: low blood/central venous pressure, pleural effusion, rapid weight gain (> 1 kg in 24 h), syncope, severe abdominal pain, venous thrombosis, anuria/acute renal failure, arrhythmia, thromboembolism, pericardial effusion, massive hydrothorax, arterial thrombosis, adult respiratory distress syndrome, sepsis, and worsening of findings [19].

The control group included patients with no evidence of OHSS of any severity after undergoing fresh ET, and the OHSS group were patients who were hospitalized and treated at the center with late moderate-to-critical OHSS.

### Statistical analysis

The incidences of the main outcome measures (obstetric complications and neonatal complications) were compared between the OHSS group and control group. First, we compared the baseline characteristics of the women according to the conception category. Potential confounders were selected based on the literature and clinical reasoning [3–10, 12–17].

The confounding baseline characteristics, including maternal age, infertility duration, body mass index (BMI), basal FSH, basal LH, antral follicle count (AFC), infertility cause, estradiol level on the hCG trigger day (pg/mL), number of oocytes retrieved from follicles with diameters ≥ 12 mm, and number of multiple gestations, were collected. We implemented a propensity score matching approach to identify the control group women who were most similar to the OHSS group women [20–25]. Propensity score matching was used to further validate the logistic regression analysis results. For the propensity score analysis, we performed one-to-four matching without replacement based on the nearest propensity scores of the OHSS and control groups [20–25]. Matching variables are presented in Table 1. The primary outcomes of our study were pregnancy outcomes, obstetric and neonatal complications, the live birth delivery rate, the miscarriage rate, gestational age at birth (weeks) and neonatal birth weight.

**Table 1:** Characteristics of the patients at baseline and outcomes of controlled ovarian hyperstimulation

Baseline characteristics	OHSS group (*n*=385)	Control group			
		Unmatched (*n*=6356)	*P* value	Matched 1:4 (*n*=1540)	*P* value
Age (years), mean ±SD	29.3±3.8	30.3±4.3	<0.001	29.4±4.0	0.585
BMI (kg/m^2^)	21.8±2.7	22.5±3.0	<0.001	21.8±2.7	0.997
Duration of infertility (years)	3.8±2.4	4.2±3.0	0.002	3.8±2.6	0.964
Baseline FSH (mIU/ml)	6.5±1.7	7.1±2.2	<0.001	6.5±1.8	0.876
Baseline LH (mIU/ml)	6.0±4.3	5.4±3.3	0.005	6.0±4.0	0.939
AFC	14.7±5.8	12.8±6.0	<0.001	14.5±6.4	0.588
Indications for IVF					
Unexplained factors (%)	10 (2.6)	320 (5.0)	0.031	42 (2.7)	0.888
Anovulatory disorders (includes PCOS)	67 (17.4)	563 (4.9)	<0.001	216 (14.0)	0.094
Tubal factors	157 (40.8)	2923 (46.0)	0.046	670 (43.5)	0.334
Endometriosis-associated	7 (1.8)	137 (2.2)	0.657	25 (1.6)	0.789
Male factors	111 (28.8)	2060 (32.4)	0.144	449 (29.2)	0.900
Pelvic inflammatory disease	3 (0.8)	56 (0.9)	1	13 (0.8)	1.000
Multiple factors	33 (8.6)	605 (9.5)	0.538	138 (9.0)	0.810
E_2_ level on hCG trigger day (pg/ml)	4484.9±1905.8	4098.5±2715.2	<0.001	4521.6±4030.0	0.795
No. of oocytes retrieved	12.2±2.9	10.5±3.8	<0.001	12.2±4.0	0.906
Multiple gestations, no. (%)	179 (46.5)	1719 (27.1)	<0.001	725 (47.0)	0.837

Note: Data are expressed as n (%) unless otherwise indicated. Plus-minus values are the mean±SD; SD: Standard deviation. Statistically significant at *P*<0.05

*OHSS* Ovarian hyperstimulation syndrome, *IVF* In vitro fertilization, *AFC* Antral follicle count, *FSH* Follicle-stimulating hormone, *LH* Luteinizing hormone, *hCG* Human chorionic gonadotropin, *BMI* Body mass index (the body mass index is the weight in kilograms divided by the square of the height in meters), *PCOS* Polycystic ovary syndrome (polycystic ovaries were defined as the presence of an antral follicle count of 12 or more or a volume of more than 10 cm^3^ in at least one ovary); Multiple factors, Infertility due to more than one infertility factor; Multiple gestations, diagnosis based on ultrasound during early pregnancy

Categorical data are represented as frequencies and percentages, and differences in these measures between the study groups were assessed by chi-square analysis with the Fisher’s exact test for expected frequencies less than 5. Continuous data are expressed as means ± standard deviations (SD) using IBM SPSS Statistics Version 22. Statistical analysis, including propensity score matching of data, was performed using R Statistical software version 3.2.5 (install.packages (“MatchIt”, R Foundation for Statistical Computing; https://www.r-project.org/). The significance testing was 2-sided, and *P* < 0.05 was considered statistically significant.

## Results

**Fig. 1:**
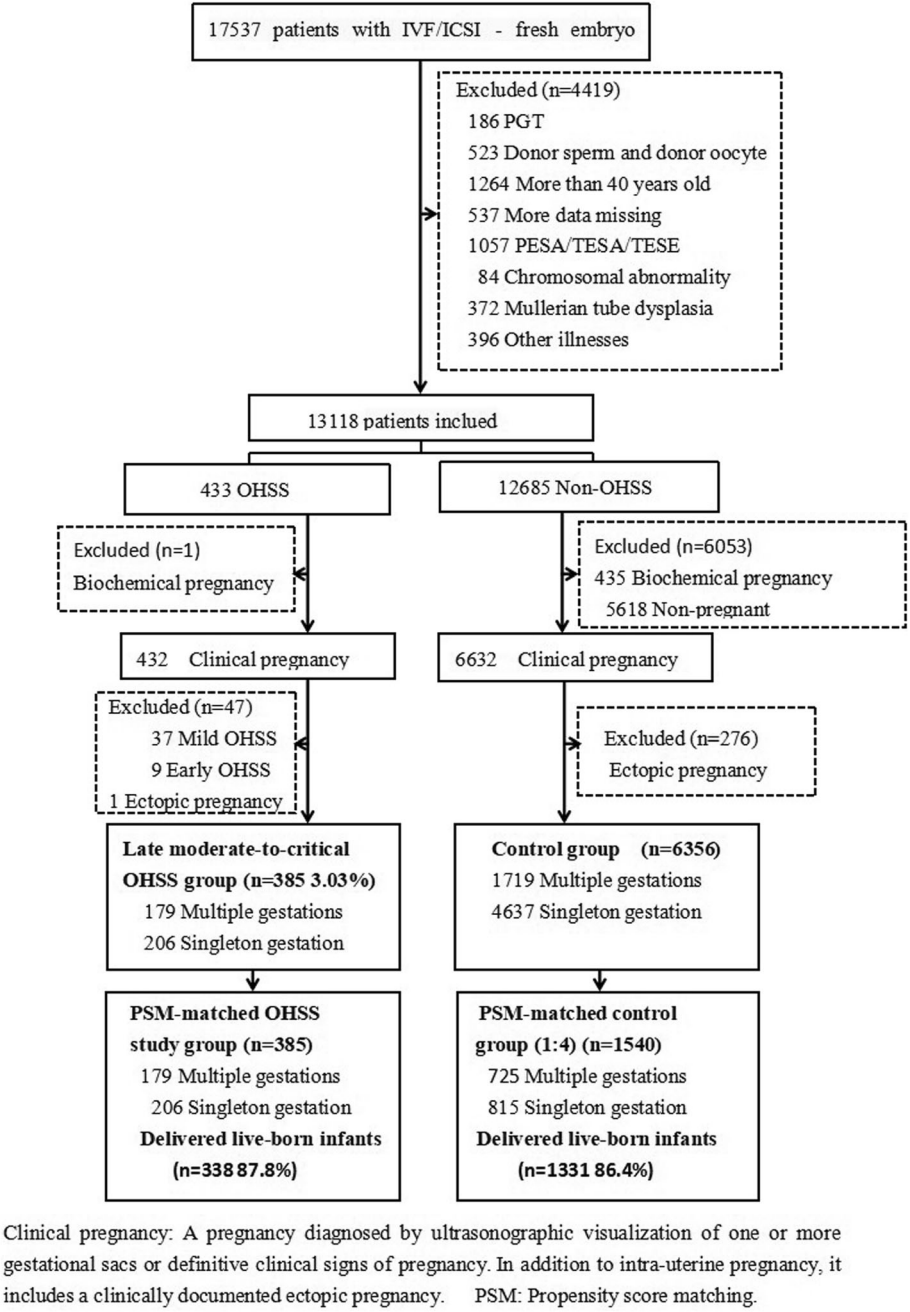
Study enrollment and outcomes. Clinical pregnancy: A pregnacy diagnosed by ultrasonographic visualization of one or more gestational sacs of definitive clinical signs of pregnancy. In addition to intra-uterine pregnancy, it includes a clinically documented ectopic pregnancy. PSM: Prospensity score matching

All 17,537 patients who met the inclusion and exclusion criteria were included, and 7,064 eligible patients who were diagnosed with clinical pregnancy after IVF/ICSI-fresh ET between June 2012 and July 2016 were evaluated. After meeting the inclusion and exclusion criteria, 6,356 patients who had no evidence of OHSS of any severity after undergoing fresh ET were allocated to the control group, and 385 (3.03%) patients who were hospitalized and treated at the center for late moderate-to-critical OHSS were allocated to the OHSS group. Then, the patients were grouped by propensity score matching as shown in the flow chart in Fig. 1.

In the OHSS group, 272 cases (70.7%) underwent fertilization by conventional IVF, and the control group included 4,646 cases (73.1%). On the day of embryonic development, the embryos were all transferred in fresh cycles. The OHSS group included the following patients: ex d3: 366 cases, 95.1%, d5/6: 18 cases, 4.9%; control group: ex d3: 5,907 cases, 90.4%, d5/6: 631 cases, 9.6%.

### Factors associated with OHSS

Compared with the control group, the patients with moderate-to-critical OHSS in our center were characterized by a young age, a low BMI, ovulation disorders or PCOS, a low basal FSH level, a higher E_2_ level on the hCG trigger day, and follicles ≥ 12 mm on the trigger day of final oocyte maturation (Table 1).

### Study patients

The basic parameters of the patients in the two groups are presented in Table 1. A propensity score matching analysis was performed with matching based on multiple maternal baseline characteristics (one-to-four), and the analysis yielded 1,540 non-OHSS patients. The baseline patient characteristics and number of multiple gestation pregnancies were similar between the two study groups (Table 1).

### Characteristics of the moderate-to-critical OHSS patients

The OHSS group comprised 385 patients (83 moderate OHSS; 289 severe OHSS; and 13 critical OHSS), with an average length of hospital stay of 12.7 ± 6.9 days.

The OHSS group included 302 (78.4%) patients who developed severe or critical OHSS. After admission, compared to the moderate group, the patients in the severe-to-critical group were hospitalized longer, and the percentage of those receiving puncture surgery was higher (9.3 ± 4.7 vs. 13.8 ± 7.2, (*P* < 0.001) and 16.9% vs. 44.0%, (*P* < 0.001), respectively). Furthermore, the HCT and WBC values in the severe-to-critical group were higher than those in the moderate group (42.0 ± 4.1 vs. 44.9 ± 5.7 (*P* < 0.001) and 14.0 ± 4.4 vs. 15.4 ± 4.8 (*P* = 0.012), respectively), as shown in Table 2. Compared with the matched control group with the same baseline characteristics, the incidence rates of miscarriages, live birth delivery, premature delivery and LBW did not significant differ between the groups. The incidence rate of obstetric complications was significantly higher in the severe and critical OHSS groups than in the matched control group; however, there were no significant differences between the moderate group and severe-to-critical group or between the moderate group and matched control group. The incidence rate of neonatal complications was significantly higher in the moderate group than in the matched control group; however, there were no significant differences between the moderate group and severe-to-critical group or between the severe-to-critical group and matched control group. The average neonatal weight in the severe-to-critical group and matched control group was significantly higher than that in the moderate group. The duration of gestation in the matched control group was significantly higher than those in the severe-to-critical group and moderate group.

**Table 2:** The characteristics of the OHSS group patients

Characteristic	Moderate group (*n* = 83)	Severe-to-critical group (*n* = 302)	Matched control group 1:4 (*n* = 1540)	P1 value1-2	P2 value1-3	P3 value2-3
E_2_ level on hCG trigger day (pg/ml)	4739.0 ± 1754.6	4415.2 ± 1935.7	/	0.171	/	/
Hospital days (days)	9.3 ± 4.7	13.8 ± 7.2	/	< 0.001	/	/
DT (days)	14.0 + 3.3	13.1 + 4.7	/	0.136	/	/
HCT (%)	42.0 ± 4.1	44.9 ± 5.7	/	< 0.001	/	/
WBC (× 109)	14.0 ± 4.4	15.4 ± 4.8	/	0.012	/	/
Albumin (g/L)	37.0 ± 4.2	36.7 ± 5.0	/	0.574	/	/
Surgical treatment, no. (%)	14/83 (16.9)	133/303 (44.0)	/	< 0.001	/	/
Miscarriages, no. (%)	9/83 (10.8)	38/302 (12.6)	209/1540 (13.6)	0.668	0.478	0.645
Live birth delivery rate, no.(%)	74/83 (89.2)	264/302 (87.4)	1331/1540 (86.4)	0.668	0.478	0.645
Obstetric complications	6/83 (7.2)	21/302 (7.0)	49/1540 (3.2)	0.931	0.058	0.002
Neonatal complications	6/108 (5.6)	12/395 (3.0)	40/1946 (2.1)	0.240	0.031	0.227
Premature delivery	15/74 (20.3)	52/264 (19.7)	262/1331 (19.7)	0.913	0.902	0.996
Average neonatal weight (g)	2698.8 ± 666.2	2828.6 ± 563.2	2853.6 ± 659.6	0.042	0.018	0.436
Singletons	41 /74 (55.4)	133/264 (44.0)	721/1331 (54.2)	0.444	0.835	0.259
Multiples	33/74 (44.6)	131/264 (49.6)	610/1331 (45.8)	0.444	0.835	0.259
Duration of gestation (weeks)	37.8 ± 2.8	38.1 ± 2.0	38.7 ± 2.1	0.435	< 0.001	0.001
LBW, no.(%)	30/108 (27.8)	98/395 (24.8)	528/1946 (27.1)	0.530	0.883	0.342

Note: Data are n (%) unless otherwise indicated. Plus-minus values are the mean ± SD; SD: Standard deviation. Statistically significant at *P* < 0.05

Statistical analysis of baseline data in the above groups revealed no difference, and the results are not shown

P1 value was Moderate group vs. Severe-to-critical group,

*DT* Days after transplantation, *OHSS* patients were hospitalized for several days after transplantation

*HCT* Red blood cell-specific volume. Normal range of values: 37–43%; pregnant: <35%

*WBC* White blood cell count. Normal range of values: 15–22 × 10^9^/l, pregnant: 6–20 × 10^9^/l

### Pregnancy and neonatal outcomes

A binary logistic regression analysis was first used to compare the perinatal outcomes of the OHSS group and unmatched control group. The pregnancy and neonatal outcomes are described in detail in Table 3.

**Table 3:** Pregnancy and neonatal outcomes of Logistic and Propensity score matching

Outcomes	OHSS group (*n* = 385)	Binary logistic regression analysis Propensity score matching			
		Unmatched (*n* = 6356)	*P* value	Matched 1:4 (*n* = 1540)	*P* value
Live birth delivery rate, no. (%)	338/385 (87.8)	5380/6356 (84.6)	0.164	1331/1540 (86.4)	0.481
Singleton, no. (%)	174/338 (51.5)	3913/5380 (72.7)	< 0.001	721/1331 (54.2)	0.376
Multiple, no. (%)	164/338 (48.5)	1467/5380 (27.3)	< 0.001	610/1331 (45.8)	0.376
Preterm delivery, no. (%)	67/338 (19.8)	1456/5380 (27.1)	< 0.001	262/1331 (19.7)	0.955
Miscarriages, no. (%)	47/385 (12.2)	976/6356 (15.4)	0.164	209/1540 (13.6)	0.481
Early miscarriages, no. (%)	23/385 (6.0)	607/6356 (9.6)	0.160	137/1540 (8.9)	0.063
Obstetric complications, no. (%)	27/385 (7.0)	414/6356 (6.5)	0.700	49/1540 (3.2)	0.001
PP, no. (%)	3/385 (0.8)	35/6356 (0.6)	0.404	5/1540 (0.3)	0.215
GDM, no. (%)	7/385 (1.8)	82/6356 (1.3)	0.178	9/1540 (0.6)	0.017
HDP, no. (%)	12/385 (3.1)	137/6356 (2.2)	0.117	34/1540 (2.2)	0.184
ICP, no. (%)	2/385 (0.5)	7/6356 (0.1)	0.056	1/1540 (0.1)	0.104
VT, no. (%)	2/385 (0.5)	0	< 0.001	0	0.040
Duration of gestation (weeks) (mean + SD)	38.0 ± 2.2	38.7 ± 2.0	0.020	38.7 ± 2.1	< 0.001
Neonatal births	503	6855		1946	
Neonatal complications, no. (%)	18/503 (3.6)	193/6855 (2.8)	0.322	40/1946 (2.1)	0.045
NICU, no. (%)	16/503 (3.2)	143/6855 (2.1)	0.103	33/1946 (1.7)	0.034
Congenital diseases, no. (%)	2/503 (0.4)	15/6855 (0.2)	0.420	1/1946 (0.1)	0.048
Average neonatal weight (g) (mean + SD)	2800.7 ± 588.6	3040.0 ± 655.3	< 0.001	2853.6 ± 659.6	0.081
LBW, no. (%)	128/503 (25.5)	1228/6855 (17.9)	< 0.001	528/1946 (27.1)	0.441

Note: Data are n (%) unless otherwise indicated. Plus-minus values are the mean±SD; *SD* Standard deviation. Statistically significant (*P*<0.05). OR, Odds ratio

Live birth delivery rate; the number of deliveries that resulted in at least one live birth, expressed per 100 cycle attempts. the denominator in our study is the number of pregnancies who were diagnosed with clinical pregnancy after IVF/ICSI- fresh ET

Premature delivery was defined as birth before 37 completed weeks and after 28 completed weeks of pregnancy

Miscarriage included early- and late-term miscarriages. Early miscarriages occurred before 12 gestational weeks, and late-term miscarriages occurred between 13 and 28 gestational weeks

Obstetric complications; *PP* Placenta previa, *GDM* Gestational diabetes mellitus, *PROM* Premature rupture of the fetal membranes, *HDP* hypertensive disorder of pregnancy, including gestational hypertension, preeclampsia, and eclampsia, *ICP* Intrahepatic cholestasis of pregnancy, *VT* Venous thrombosis

Neonatal complications included prematurity, extremely low birth weight, perinatal asphyxia, major birth defects, sepsis, neonatal jaundice, and infant respiratory distress syndrome due to immaturity of the lungs

NICU, neonatal intensive care unit, which concentrates on the care of premature babies and sick newborns, due to extreme low birth weight (LBW), perinatal asphyxia, major birth defects, sepsis, neonatal jaundice, and infant respiratory distress syndrome due to immaturity of the lungs and other complications

Other neonatal complications: One neonatal death occurred in the NICU, and two congenital diseases occurred in the OHSS group. In the matched non-OHSS group, one death occurred within one year after birth, and one congenital disease, one chromosomal abnormality and five congenital diseases were present

*LBW* Low birth weight (birth weight <2500 g)

Threatened abortion refers to a small amount of vaginal bleeding, often dark red or bloody leukorrhea and accompanying paroxysmal abdominal pain or lower back pain in the absence of pregnancy discharge at <28 weeks of pregnancy

All patients in the two groups had a clinical pregnancy, and the abortion rate of the unmatched control group was higher than that of the OHSS group, but there was no significant difference (12.2% vs. 15.4%, *P* = 0.098). There was no difference in the abortion rate between the two groups after matching (12.2% vs. 13.6%, *P* = 0.481).

Before matching, several parameters differed between the patients in the OHSS group and the controls. However, after propensity score matching, the perinatal outcomes of the OHSS group and matched control group were compared. The live birth singleton delivery rate and preterm delivery rate did not significantly differ between the two groups. The incidence rate of obstetric complications of concern was significantly higher in the OHSS group than in the matched control group (7.0% vs. 3.2%; *P* = 0.001). Moreover, the GDM and venous thrombosis (VT) rates were higher in the OHSS group than in the matched control group (1.8% vs. 0.6%; *P* = 0.017 and 0.5% vs. 0%; *P* = 0.04, respectively).

The incidence rates of neonatal complications and number of neonates admitted to the NICU were significantly higher in the OHSS group than in the matched control group (3.6% vs. 2.1%; *P* = 0.045 and 3.2% vs. 1.7%; *P* = 0.034, respectively). The duration of gestation in the matched non-OHSS group was significantly higher than that in the OHSS group (38.7 ± 2.1 vs. 38.0 ± 2.2; *P* < 0.001). However, no significant between-group differences were evident in the average neonatal weight (g) or LBW (2800.7 ± 588.6 vs. 2853.6 ± 659.6; *P* = 0.081 and 25.5% vs. 27.1%; *P* = 0.441, respectively).

## Discussion

The occurrence of OHSS-associated hospitalizations increases the economic burden and affects patient mental wellbeing after IVF-ET [26]. However, different races, regions, hospitals or research methods may affect the impact of OHSS on pregnancy outcomes; for instance, the baseline characteristics or severity of OHSS in patients may affect interpretation of the results during the course of clinical research. The pregnancy outcomes of pregnancies effected by OHSS have not yet been investigated thoroughly, and further studies are needed [8, 12]. The results of our data in the OHSS group and unmatched control group showed that the rates of multiple live birth deliveries and LBW were significantly higher in the OHSS group after eliminating the impact of multiple pregnancies and nine baseline characteristics on perinatal complications using propensity score matching. Furthermore, the results of our data in the OHSS group and matched control group showed that the incidence rates of obstetric complications and neonatal complications were significantly higher in the OHSS group than in the control group, including the incidence of GDM, VT, congenital disorders and neonatal NICU hospitalization. No significant between-group differences in the rates of preterm delivery, miscarriage, and early miscarriage were observed.

In our study, obstetric complications were significantly higher in the OHSS group than in the control group, but the incidence rates of PP, HDP, and ICP were not increased after OHSS, and the rates were consistent with previously reported post-IVF rates [27, 28]. Our results are similar to several previous reports that assessed this outcome [12, 14]. A previous symposium by Raziel et al. in 2009 and a previous case-control study indicated that the pregnancy rate is increased in OHSS patients and that the incidence rates of multiple pregnancies, GDM, premature birth, and LBW infants are significantly higher in OHSS patients [8]. We observed thrombosis only in the OHSS group. These results are rather inconsistent with previous findings because the obstetric complications examined here were not evaluated in previous studies [8, 13, 14].

A previous case-control study reported that the hospitalization duration of OHSS patients was positively related to increases in the rate of miscarriage and that OHSS hospitalization was not conducive to pregnancy or continued pregnancy in patients who underwent IVF [18]. It is possible that the occurrence and treatment of OHSS does not affect the abortion rate. At admission, the HCT and WBC values were positively correlated with the degree of OHSS and patient symptoms and were associated with an increased rate of surgical treatment. The number of hospital stays in the severe-critical group was higher than that in the moderate group, and the number of obstetric complications was decreased; however, the number of neonatal complications were increased, as shown in Table 2. The severity of OHSS increased the incidence rates of obstetrical complications and preterm delivery but had no effect on neonatal complications.

## Conclusions

After eliminating the effects of confounding factors, late moderate-to-critical OHSS could reduce the gestational time and increase the number of obstetric complications and neonatal complications, including the incidence of GDM, VT, congenital disorders and neonatal NICU hospitalization. However, the live birth rate, average neonatal weight and incidence rates of premature delivery, miscarriage, early abortion, HDP, PP, ICP, and LBW were not statistically different between the two groups.

## Data Availability

All data supporting the conclusions of this article are included.

## Abbreviations

IVF: In vitro fertilization
ICSI: Intracytoplasmic sperm injection
BMI: Body mass index
hCG: Human chorionic gonadotropin
HDP: Hypertensive disorder of pregnancy
GDM: Gestational diabetes mellitus
LBW: Low birth weight
OHSS: Ovarian hyperstimulation syndrome
